# Supplementary material for: Uptake and Distribution of Administered Bone Marrow Mesenchymal Stem Cell Extracellular Vesicles in Retina
Source: Cells. 2021 Mar 25;10(4):730. doi: 10.3390/cells10040730 (PMC8064505; doi:10.3390/cells10040730)
Supplement: Supplementary file 1 [file cells-10-00730-s001.zip › SUPPL/Figure Legends for Supplemental Video Figures for Mathew.pdf]

Figure Legends for Supplemental Video Figures for Mathew, B et al, Cells 2021

Suppl. Video Figure S1: Astrocytes stained with anti-vimentin are seen in red, EVs in green, and DAPI in blue. The movie shows the cells in a 3-D view rotated around the Y-axis plane, which enhances visualization of the EVs inside the cells.

Suppl. Video Figure S2: Microglia stained with anti-CD11b/c are seen in red, EVs in green, and DAPI in blue. The movie shows the cells in a 3-D view rotated around the Y-axis plane, which enhances visualization of the EVs inside the cells.

Suppl. Video Figure S3: Retinal neurons stained with α-tubulin are seen in red, RGCs stained with anti-Brn3a in magenta, EVs in green, and DAPI in blue. The movie shows the cells in a 3-D view rotated around the Y-axis plane, which enhances visualization of the EVs inside the cells. The white staining inside cells is overlap of magenta and green (EVs inside RGCs), and yellow staining is overlap of red and green, which can also be seen along neurite projections.

.
