# Supplementary material for: Uptake and Distribution of Administered Bone Marrow Mesenchymal Stem Cell Extracellular Vesicles in Retina
Source: Cells. 2021 Mar 25;10(4):730. doi: 10.3390/cells10040730 (PMC8064505; doi:10.3390/cells10040730)
Supplement: Supplementary file 1 [file cells-10-00730-s001.zip › SUPPL/Suppl Tables 1 and 2.pdf]

Name	Manufacturer	Type	Catalog #	Dilution
α-Tubulin	Sigma-Aldrich	Mouse monoclonal	T6199	1:500
β-Tubulin III	Sigma-Aldrich	Mouse monoclonal	T8660	1:1000
Brn-3a	Santa Cruz	Mouse monoclonal	sc-8429 AF647	1:500
CD11b/c	BD Pharmingen	Mouse monoclonal	550299	1:500
GFAP	Agilent Technologies	Rabbit polyclonal	Z033429-2	1:1000
IBA-1	Novus Biologicals	Goat polyclonal	NB100-1028	1:750
RBPMS	Proteintech	Rabbit polyclonal	15187-1-AP	1:500
Vimentin	Santa Cruz	Mouse monoclonal	sc-6260	1:1000

Suppl. Table S1: Antibodies used in the study

**Brn3a/BT3**							
Pearson's R	TM1	TM2	Li's ICQ	Spearman Rank	Spearman T-Stat	Costes P	Kendall's Tau
0.68	0.7523	0.795	0.261	0.635	683.4769	1	0.4609
**IBA-1**							
Pearson's R	TM1	TM2	Li's ICQ	Spearman Rank	Spearman T-Stat	Costes P	Kendall's Tau
0.51	0.4472	0.8023	0.272	0.50195	214.4017	1	0.4316
**Vimentin**							
Pearson's R	TM1	TM2	Li's ICQ	Spearman Rank	Spearman T-Stat	Costes P	Kendall's Tau
0.38	0.0869	0.7427	0.155	0.1358	140.3122	1	0.1172

Suppl. Table S2: Analaysis of co-localization in flatmounts in vivo. See Methods for details. Shown here are the co-localization parameters for green EVs and Brn3a/BT3 for RGCs, IBA-1 for microglia, and vimentin for astrocytes.
